# Empowering local research ethics review of antibacterial mass administration research

**DOI:** 10.1186/s40249-022-01031-6

**Published:** 2022-09-28

**Authors:** Nelson K. Sewankambo, Paul Kutyabami

**Affiliations:** 1 Department of Medicine, School of Medicine, College of Health Sciences, Makerere University, P. O. Box 7072, Kampala, Uganda; 2 Department of Pharmacy, School of Health Sciences, College of Health Sciences, Makerere University, P. O. Box 7072, Kampala, Uganda

**Keywords:** Antibiotic, Mass drug administration, Ethics Review Committee, Ethics Review, Institutional Review Board, Empowerment, Guidance, Trials

## Abstract

**Background:**

Recent studies using mass drug administration (MDA) of antibiotics to entire communities have focused global attention on the unique ethical challenges of MDA of antibiotics in research and public health interventions. However, there is no specific guidance for Research Ethics Committees (RECs) or Institutional Review Boards (IRBs) to review such trials. We surveyed the literature to identify the unique ethical challenges and to strengthen the competencies of RECs or IRBs in low- and middle-income countries (LMICs) in their ethical reviews of these trials.

**Methods:**

We employed a desk review. We searched PubMed, Web of Science, and Google Scholar, combining terms for “mass drug administration” with terms for “research ethics committees,” “institutional review boards,” and “ethics.” We reviewed citations of search results to retrieve additional articles. Only articles published and indexed in the above databases up to 6 January 2022 in English were included. Abstracts (without full articles), books and articles that had exclusive veterinary and environmental focus were excluded. We synthesized the literature to identify particularly challenging ethical issues relevant to antibacterial MDA trials in LMICs.

**Results:**

The most challenging ethical issues can be categorised into four broad domains: determining the social value of MDA, assessing risks and benefits, engaging all stakeholders meaningfully, and study design-related ethical challenges. These four domains interact and impact each other. Together, they reveal the need for RECs/IRBs to review MDA studies through a broader lens than that of clinical trials per se. From our findings, we propose a framework to guide the RECs and IRBs in LMICs to perform the initial and continuing review of antibiotic MDA trials. We also recommend strengthening the competencies of LMIC RECs or IRBs through ongoing training and collaboration with RECs or IRBs from high-income countries.

**Conclusions:**

REC/IRB review of research using MDA of antibiotics plays a critical role in assuring the ethical conduct of MDA studies. Local RECs/IRBs should be empowered to review MDA studies comprehensively and competently in order to advance scientific knowledge about MDA and promote improved global health.

## Background

Ethics review in health research is the assessment of the ethical quality and processes of proposed, ongoing, and completed studies. Research Ethics Committees (RECs)—also known as Institutional Review Boards (IRBs)—play a key role in the oversight of research; they engage in research ethics review, monitoring, and regulation aimed at protecting individual research participants and the integrity of the research enterprise. Since their inception in the 1950s [1], the research ecosystem has been evolving, including the emergence of new trial designs and methods [2–7]. These developments pose both theoretical and practical novel challenges for ethical review because of the increased complexity of expertise required by RECs to perform their duties optimally [2–8].

Mass drug administration (MDA) trials represent one such development. MDA is not new. The control and elimination of some helminthic, parasitic, and bacterial diseases in low- and middle-income countries (LMICs) hinge on MDA use [9–17]. It is widely recognized that antimicrobial use (which target microorganism generally)—most especially of antibiotics (which target bacteria specifically)—must be judicious to prevent or minimize the development and spread of anti-microbial resistance and other unintended consequences [18–20].

The placebo-controlled, cluster-randomised Macrolides Oraux pour Reduire les Deces avec un Oeil sur la Resistance (MORDOR)-I trial, which showed a significant reduction in mortality among children aged less than 5 years given azithromycin MDA in one (Niger) of the three study countries (Mali and Tanzania were the others) [19], has focused new attention on MDA trials. Although the drug’s mechanism of reducing childhood mortality is unknown, largely on the basis of MORDOR, the World Health Organization (WHO) provisionally recommended universal biannual mass administration of azithromycin for children aged 1–11 months in countries with high child mortality and where existing child survival interventions are also strengthened [21].

Mass administration of this antibiotic has begun to inspire ethical debate [22], but MORDOR is not the only relevant MDA study. Other azithromycin MDA trials are ongoing, such as the Azithromycin Pour la Vie Des Enfants au Niger—Implémentation et Recherche (AVENIR) study in Niger [23] and the Large-scale Assessment of the Key health-promoting Activities of two New mass drug administration regimens with Azithromycin (LAKANA) [24] and the Sauver Avec l'Azithromycine en Traitant Les Femmes Enceintes et Les Enfant (SANTE) studies in Mali [25], to elucidate further the safety, efficacy, mechanism, and long-term effects of azithromycin MDA. The scope of these studies is impressive. The AVENIR study is a double-masked cluster-randomized placebo-controlled response-adaptive large sample trial that targeted 3350 communities in Niger [23]. The LAKANA study planned to include 100,000 infants in 830 villages to date [24] and is a cluster-randomized placebo-controlled, double-blinded, parallel-group, three-arm trial with adaptive design. The azithromycin intervention is delivered in the context of a seasonal malaria chemoprevention (SMC) program. AVENIR and LAKANA are also examples of the adoption of alternative trial designs in MDA research by combining intervention delivery with implementation science. Additionally in Mali the SANTE trial is conducted to determine the effectiveness of oral azithromycin in preventing stillbirths and infant mortality [25].

Recognizing the key role RECs/IRBs play in research review, we reviewed the literature to provide practical guidance for REC reviews of MDA research in LMICs. Although MDA research shares many research ethics concepts and issues with global health research generally, we intentionally focus only on those issues that have special relevance and are particularly challenging in antibiotic MDA research proposals.

## Method

Data for this review were identified using a search of PubMed, Web of Science and Google Scholar, combining terms for “mass drug administration” with terms for “research ethics committees,” “institutional review boards,” and “ethics.” We also did a citation search of returned articles and retrieved additional articles. Only articles published and indexed in the above databases up to 6 January 2022 in English were included and there was no beginning date cutoff. The inclusion criteria were: (i) original empirical research articles, editorials, and commentaries that (ii) addressed antibacterial MDA for human health and that (iii) discussed the ethics review of such studies by RECs or IRBs. We excluded abstracts (without full articles), books and articles that had exclusive veterinary and environmental focus. The azithromycin MDA literature was central to this study. We synthesized the literature to identify the ethical issues uniquely relevant to antibacterial MDA trials in LMICs and to which RECs should pay particular attention beyond the usual review processes. Each of the two researchers NKS and PK independently reviewed the articles from the search and identified the most commonly cited ethical challenges which the authors considered to be most important and of greatest impact on society and the environment and either how they can be identified and prevented to become major challenges or addressed when they occurred/identified. We met over several weeks and compared our individual findings and four domains of ethical issues emerged and are the focus of this paper.

## Results

Our review revealed that antibiotic MDA research is characterised by complex ethical issues which can be difficult to resolve because of value conflicts, uncertainty surrounding the societal impact and implications of research, environmental concerns, and persistent unknowns about antibiotic MDA, among other issues. In general, for review of antibiotic MDA research, RECs should conduct scheduled annual reviews, and in addition they should consider reviewing these studies more frequently. Depending on the study specifics and timeline, for example, RECs can require continuing review every 3–6 months, after a set number of subjects are enrolled or doses given, or on an ad hoc basis. RECs should also follow the same approach as they do for any other health research proposals using the principles for protection of human research participants. Like for all studies, for example, RECs should ensure that MDA is “responsive” to the health needs or priorities of the communities or populations where the research will be conducted [26]. In addition, RECs must assess whether the trial design adequately justified both scientifically and ethically to answer the study question. Frameworks exist to help RECs do this, but these frameworks have not been applied to the unique issues of MDA [27]. They should increase their attention to the alternative trial designs that are increasingly adopted by researchers [28–31].

Whether or not ethical issues are unique to MDA studies, some take on unique dimensions or may be more salient in the antibiotic MDA context. Before presenting our main findings, we wish to make a special note regarding informed consent. Informed consent can be challenging in MDA studies at both the individual and community levels. Individually, participants must be informed of the risks and benefits of participation, both short- and long-term. However, this can be challenging when MDA research occurs alongside existing public health interventions (such as standard vaccine campaigns or other mass drug administrations targeting diseases, such as malaria). At the community level, given that the whole community may be involved in or affected by the trial [e.g., due to antimicrobial resistance (AMR), which is not limited to trial participants] community involvement is essential. In addition, cultural context—e.g., different cultural norms around consent or antibiotics in general, when antibiotics may be easily accessible without a prescription—is critical to consider. Nevertheless, we found that existing guidance regarding consent in LMICs is adequate to support REC review of this important issue; we therefore discuss informed consent below only where it relates to other critical ethical issues.

In what follows, we present those four broad domains (social value, risk–benefit ratio, stakeholder engagement, and study design related ethical challenges) that our review found to be most important for RECs to consider in MDA research (Box 1). These domains form the main sections of this paper. Table 1 summarises select details for each domain, emphasizing questions that the REC members should ask at initial and follow-up review.

**Table 1:** The following key ethical questions should guide the initial and continuing review processes throughout the research cycle

Domain	Dimension	Questions to ask during initial review	Questions to ask during a continuing review
Social value	The overall value of the anticipated knowledge gain and change in health and wellbeing of groups of people or communities attributed to the intervention	How well are the objectives aligned to address an important knowledge gap (or scientific equipoise) for which antibacterial MDA is a potential solution? How appropriate are the selected interventions—both MDA and others in the study—for achieving real social value?	What new research findings and research ideas have emerged that affect social value? What circumstances would make it necessary to have design change, and/or additional research support to enhance social value?
	Benefits (including *ancillary benefits*, i.e., benefits outside the scope of the study itself)	What is the magnitude, likelihood, and nature of the benefits (i.e., well-being) accruing from the intervention and from benefits outside the specific research target to individuals and the community and stakeholders?	What benefits (including ancillary ones) have been realized?
	Additional interventions	What additional non-MDA interventions are or should be included to modify, complement or maximize social value?	What additional interventions have been implemented?
	Assumptions regarding human and social responses to the MDA intervention	What study- and context-specific assumptions are being made that could affect the successful implementation of the MDA intervention?	Were the assumptions correct, and how do they need to change to facilitate MDA implementation?
	Implementation of MDA (e.g., using existing health system or creating a parallel system)	What strategy will be used to deliver the MDA (e.g., via existing health or public health systems, or not) and how will this affect overall social value (including sustainability)?	What impact has the delivery strategy had on the health system?
Assessment of risks and burdens	Identifying risks and burdens arising from the antibacterial agent	What risks and burdens are anticipated to arise from the antibacterial drugs, including adverse events (AEs) and AMR? What mechanisms are proposed to monitor and measure AEs – including AMR and other drug-related risks—and burdens? How will both short- and long-term risks be monitored, for both enrolled individuals and the broader community?	What anticipated and unanticipated AEs were more or less frequent than expected? How complete was the reporting of AEs to the relevant Regulatory Authorities? What impact did the anticipated and unanticipated AEs have on the risk–benefit balance?
	Risk and harms arising from adverse human and social responses to the intervention (*pragmatic risks*)	What is the magnitude, likelihood, and nature of the risks and harms anticipated to arise from human and social responses to the MDA and non-MDA interventions in the study?	How have the anticipated and unanticipated human and social responses affecting implementation been captured, and how should they be managed, moving forward?
	Risk minimization	What proactive measures are proposed to minimize AEs, AMRs, and other drug-related risks and burdens? What measures are proposed to minimize risk and harms arising from human and social responses to MDA?	What measures for risk minimization were used?
	Ethical issues concerning post-trial surveillance for AMR and other AEs	What ethical challenges will be addressed by the post-study surveillance plan for monitoring the emergence and spread of AMR and other MDA-related AEs (and for how long)?	What new relevant ethical challenges and solutions have been identified for continued monitoring and emergence of AMR and other MDA-related AEs?
Stakeholder engagement	Meaningful stakeholder engagement and community ownership of the study	How will the people and other entities likely affected by MDA research and its results be identified in order to engage them?	What were the challenges in defining stakeholders and engaging them? How were their interests and concerns addressed?
		What are the study roles and responsibilities of the different stakeholders engaged?	What were the challenges and lessons learned in implementing the engagement plan?
	Implementation plan	How realistic is the plan (goals, objectives; activities, adequate finances, and human resources?	How is engagement with stakeholders being evaluated (process, outcomes of engagement, development of mutual trust, respect, transparency)?
	Minimization of undue influence or biases in engagement	How will undue influence or biases among engaged stakeholders be minimized and monitored? What controls are in the engagement plan and study design to mitigate ethical concerns that may arise from the potential influence of research partners from HICs?	Which ethical concerns surrounding HIC funding and researchers were formally or informally reported and how were they resolved? How well are the controls working and which improvements need to be put in place?
	Post-MDA engagement	How will study communities and other stakeholders be engaged to prepare them for uptake of the MDA intervention in the post-study follow-up and scale-up?	How did study communities begin to prepare themselves for sustainability?
		Are there plans for future financing of an MDA intervention program?	What agreements with policymakers and others are in place for a financing plan to support the scaleup of the MDA if the study findings are positive?
Study design-related ethical challenges	Pragmatic and other alternative trial designs are commonly used in MDA studies	Which are the most relevant ethical challenges arising from the particular study design?	To maintain high ethical quality, is the study design still ethically appropriate, or should protocol amendments be considered?
		How are study participants defined in the study (e.g., as only those who take the MDA drug, or others)? How is minimal risk defined, and which potential risks and benefits will be disclosed to participants?	What were the challenges in the consent process? If the study was classified as minimal risk, does it still meet minimal risk criteria?
		If a waiver of informed consent, its documentation, or other alterations to consent is included, is it justified?	What reasons were given by different types of refusals to participate in the whole or part of the study?
	MDA trials are more akin to public health interventions; a straight-forward clinical trial ethics review is not ideal for review	How well are the nexus of public health, research, and clinical ethics challenges, including human rights, addressed? Does the IRB/REC have sufficient expertise to evaluate the study rigorously?	Have any new ethical challengers emerged that pose capacity challenges for the LMIC REC to review study progress satisfactorily? How can the capacity of HIC IRB be harnessed to strengthen LMIC REC capacity?
	Choice of intervention in the comparator arm	What is the ethical justification for the intervention in the control arm (e.g., placebo)? What are the plans to ensure that contamination is minimized?	Does the justification of the control arm intervention still hold based on new scientific knowledge?

*AEs* Adverse events, *AMR* Antimicrobial resistance, *HIC* High-income country, *IRB* Institutional Review LMIC, *LMIC* Low- and middle-income countries, *MDA* Mass drug administration, *REC* Research Ethics Committee

Box 1. Core domains of ethical challenges for REC’s review in antibiotic MDA research

**Table:** 

• **Social value**: Probability of sufficient social value to justify exposing whole communities to known and unknown risks without prospect of direct benefit to uninfected participants
• **Risk-benefit ratio**: A ratio that takes into account current and future generations in a context of unknown risk levels for adverse events like the undetermined magnitude, speed and spread of AMR and/or durability of benefits, and their development pathways
• **Meaningful stakeholder engagement**: How stakeholders who are within and beyond study communities act is a major determinant of the eventual utility of the study findings
• **Study design-related challenges**: These arise from mass use of antibiotic(s) and the large pragmatic cluster randomised trial design leading to challenging ethical issues

### RECs should evaluate whether MDA studies promote social value

The central purpose of conducting trials is to generate knowledge and measures to promote health and wellbeing of individuals and their communities [26, 32]. REC evaluation of social value must ensure that trials have sufficient value to society to justify both the risks to participants and society as a whole and the resources needed to conduct them [33, 34]. In the long history of research ethics, social value has only recently been systematically evaluated [35–37]. On one account, to understand social value, RECs need to consider the knowledge to be gained, the likelihood of attaining it, and how that new knowledge affects stakeholder decisions (e.g., local and national policymakers) [38].

Ordinarily RECs are comfortable considering direct benefits and risks to participants [39]. Such benefits (e.g., the prospect of improved health or well-being) are tangible and clear, and there are available REC guidelines for their evaluation [40]. However, assessing social value requires attention to a far broader context, requiring an assessment at least of the uncertainty of anticipated and unanticipated direct and indirect benefits and risks for individuals, communities, and society [36]. Social value of trials should be evaluated against the anticipated risks and burdens at the initial review and the unanticipated risks and burdens that emerge during implementation during continuing review.

The challenge of MDA research studies is that it is precisely the social value of MDA—particularly MDA for the sake of reducing child mortality—that is in question, yet there are no clear guidelines for RECs to assess social value of MDA [35, 41]. Moreover, social value may differ among communities and is not one single, nationwide assessment; there may be significant variation in the anticipated social value for different groups, due to differences in health needs and expectations from the intervention [34]. Health needs and expectations among community groups are shaped by their understanding of the disease, its impact on quality of life and wellbeing, and the anticipated efficacy and safety of the intervention compared to other available alternatives [42].

Social value is therefore context specific and must be assessed in comparison to other potential interventions, particularly in settings where certain resources are limited. RECs should be aware of the social, cultural, political and economic factors that influence implementation of MDA research and consequently affect social value [42–44]. Such factors can lead to differences in beliefs about the disease and efficacy and safety of the intervention. For example, in some communities globally, there are beliefs that injectable and coloured medicines are more efficacious than oral and white medicines and hence are preferred for conditions deemed severe [45]. In others, conditions that outsiders may perceive as requiring urgent action may not be perceived as such by the individuals experiencing them; these individuals may have adapted to their situation [46], therefore viewing the intervention as too late or of lower priority and therefore unnecessary [47]. Such circumstances can affect how social value is assessed, and must be taken into account when RECs consider social value—a basic requirement for ethical research.

As such, when evaluating the social value of MDA trials, which are frequently conducted across multiple institutions and countries, local RECs have a special role to play. Researchers should be expected to explicitly have justified the social value of the study to the local community in the protocol. Based on that, RECs should carefully evaluate social value reflecting on their local knowledge, experience and context. For RECs to aptly do this, they need to consider the aims and procedures of the trial, and determine if they are sufficient to actually support the anticipated improvement in health and wellbeing.

RECs may also consider whether attaining sufficient social value requires MDA trials to include complementary interventions like environmental hygiene and community health education, in order to maximize or sustain the desired improvements in health and well-being (e.g., decreased childhood mortality). For example WHO recommended the SAFE strategy for elimination of trachoma by 2020; antibiotics are only one part of this [48]. Similarly, the WHO Guidelines recommend MDA for childhood mortality, only when existing child survival interventions are concurrently strengthened [49]. Communities may find these interventions to be more socially valuable than, say, the laboratory facilities necessary to monitor AMR from an MDA research program or the MDA itself.

Finally, researchers may have assumptions about social value of the MDA trial, i.e., that (1) the medicine will obviously be accepted by the community and appropriately administered; (2) a substantial proportion of population will have the medicine administered to achieve the desired and eventual herd effect; and (3) the medicine administered will continue to be effective in the real-world, post-trial, with acceptable safety profile. RECs have the responsibility to ensure that these assumptions hold true.

### RECs should evaluate comprehensively the risks and benefits of MDA studies

Generally, assessment of risks and burdens probes two aspects; the probability of occurrence of the potential risks and burdens, and their magnitude or severity if they occur [50, 51]. Risks and burdens can be anticipated or unanticipated, either because they have or have not been experienced in previous studies. Assessment of risk and burden require RECs to weigh the magnitude and severity of harm and discomfort anticipated in research against what is ordinarily encountered in daily life during performance of routine or psychological examinations [50].

MDA trials with antibiotics present five peculiar aspects in this assessment: (1) The involvement of large numbers of individuals receiving medicines is likely to manifest with a greater surge in the anticipated and unanticipated risks and burdens than previously experienced including hastened development of AMR; even rare adverse events, such as choking on tablets, might occur in such large studies [52]. (2) Some of these risks accrue to bystanders, i.e., individuals and communities who did not participate in the study, including future generations (e.g., if antibiotic resistance were to spread) [53]. (3) The administration of antibiotics to individuals who do not feel ill and do not necessarily see the need to take antibiotics for a “cure” complicates how risks are perceived. (4) The involvement of the entire community may arouse adverse social responses to the implementation of the MDA trial that could impact prospects of attaining the social value [54]. (5) Implementation of MDA may have unintended consequences for service delivery systems especially when MDA is implemented through existing community resources [46].

Since MDA trials usually administer drugs of proven efficacy and safety that are used as standard in clinical practice [55], RECs might consider them to be of low risk or of no more than minimal risk. Such an assessment may be accurate at the level of the individual participants. However, mass administration of same drugs may confer risk more than what is ordinarily encountered in daily life. RECs should consider the likelihood of a surge in anticipated and unanticipated adverse drug reactions (ADRs), hastening of AMR development, and provoking adverse social responses to trial interventions (e.g., a prohibition of public defecation in setting where this is the cultural norm) that need to be closely monitored and appropriately mitigated. Trial interventions that contravene usual cultural practices, even if well-intended, may be resisted by the community leading to hostility against implementation of the MDA [56, 57]. Because such risk is different from risks to individual trial participants, it is known as a “pragmatic risk” that could undermine current and future research studies. RECs must ensure that mechanisms to monitor and mitigate such risks and burden are well elaborated in the protocol at the initial review and implemented at the continuing review to ensure overall positive balance in social value.

During a typical MDA trial, the entire community receives the drugs, with few exceptions (e.g., pregnant women, neonates and people known to be hyper sensitive to the drug). In MDA research, this will include individuals without known disease. The uninfected individuals may see no reason to take medicines and, on the contrary, may worry that, taking the medicines my make them sick if they experience ADRs [45, 46]. Giving medicines to uninfected individuals as prophylaxis can be justified based upon the anticipated social value to current and future individuals and communities.

Although RECs are typically thought of as constrained to assessing risks of studies narrowly—so as not to include, for example, longer-term, uncertain risks or the social consequences of research findings—MDA studies involving antibiotics reveal this constraint to be problematic. In particular, if AMR is a major risk of MDA studies to individuals and communities both now and in the future, it provides a critical link for RECs to therefore consider AMR in the risk-benefit assessment of MDA research.

Finally, MDA can present risks to the broader health sector. Community wide interventions like MDA trials are usually costly to implement using parallel service delivery arrangements. It is common for researchers to consider using resource of existing service delivery systems like in health, education, and security sectors to reduce costs. This may divert or overstrain resources in these vital sectors, compromising service delivery. Similarly, resource limited settings are usually faced with limited skilled people to take on roles in implementation of MDA trials. Trials could either temporarily take skilled staff from existing service delivery systems or use unskilled staff. Taking skilled staff off their regular work affects service delivery while using unskilled staff affects implementation of the trials. It is necessary that RECs probe how the MDA will be implemented to determine the likely effect on services delivery and efficacy of implementation.

### RECs should evaluate meaningful engagement of all stakeholders affected by MDA

Community engagement is identified in the CIOMS ethical guidelines as an ethically important practice in research [26]. In addition, the principle of respect for communities proposed by Weijer [58] requires “researchers to respect communal values, protect and empower social institutions, and, where relevant to respect the decisions of legitimate communal authorities”.

Our review found that, in antibiotic MDA trials, stakeholder engagement is a more comprehensive and appropriate concept than community engagement. Stakeholders are individuals or group actors (for example health service user groups, patient groups, research team, study communities, national or sub-national governments, non-governmental organisations, civil society, private actors, international organisations, funders, service providers, patients, and the media) who can influence or be affected by the research processes or outcomes [59]. The effectiveness of an antibiotic MDA intervention to obtain sufficient social value is dependent on the population’s collective engagement and actions of all stakeholders and not only study communities [60–63]. The potential impact of these trials may be far reaching, affecting study and non-study participants beyond national boarders, agricultural animals, the environment, and future generations [53].

The REC review should ensure that the investigators describe how all legitimate study stakeholders are to be identified and engagement extended to priority groups that are likely to be most affected from research risks/harm and benefits [59, 64]. They should be engaged as appropriate from the protocol development stage all through the research cycle. The REC, however, should take into consideration the available budgetary resources and not impose unreasonable demands for engagement activities [65].

The research team should have a stakeholder engagement plan. The RECs should review the plan, its appropriateness and likelihood that the ethical value proposition premised on meaningful engagement of stakeholders will be achieved. The plan has a defined engagement strategy, activities, clarification of different stakeholder obligations during and after study completion, expected outcomes, evaluation plan and adequate resources. There is no one size fits all plan because implementation activities may differ from one study to another and need to be customised to account for uniqueness of context. There is a range of activities that occur along a spectrum that extends from informing, consulting, involving, collaborating and sharing partnership and leadership [64, 66].

Researchers must provide space to listen to individuals and stakeholders, hold deliberations which facilitate understanding of the study by all various actors, and also enable researchers to understand stakeholders’ concerns and their interests [64, 66]. This will also improve transparency and accountability on both sides, the researchers’ understanding of the stakeholder’s socio-cultural, political context, and the stakeholders’ perceived social value [42–44]. The ethical responsibilities can be successfully implemented based on trust, mutually respectful relationships between researchers and stakeholders, adherence to the transparency principle, and making unbiased accountable decisions [66, 67]. Communication with communities and other stakeholders can make or break the trial.

RECs should also review and approve communication messages prior to their use in the communities and with other stakeholders or the wider public. These may be in the form of information sheets, pamphlets, posters, drama, radio or television (TV) broadcasts, videos, and social media content. REC members can find details concerning engagement practicalities discussed elsewhere [60–66] including some national guidelines.

The desired engagement outcomes that RECs should expect to be addressed are empowerment of the different stakeholders; ownership of the study by stakeholders; active engagement throughout study activities; and high compliance with the antibiotic(s) regimen and study guidelines. In addition, engagement will promote understanding by all parties of the known study burdens and benefits, identification of the non-obvious risks/burdens and benefits, and their potential distribution between individuals, communities and society [66]. This will enhance risk–benefit assessment discussed above during the entire research cycle.

During initial and continuing reviews RECs should assess whether the study team achieved meaningful engagement which will enhance post-trial availability of interventions and stakeholders’ uptake and intervention scale-up. Sustained engagement with local or national and international policy makers, drug manufacturers and funding agencies will facilitate creation of funding streams for a sustainable adequate supply of the post-study medication, consistent with CIOMS guidelines [26]. This enhances social value of the study [41, 68]. A practical challenge for RECs and researchers is that in LMICs it is often not clear how broadly the RECs should apply this requirement.

Lastly, it is important for RECs to identify during the review process the likely potential undue influence on the study design, implementation and reporting of results by the more influential stakeholders (like study sponsors/funders, strong research groups, policy makers and political or lobby groups). There should be mechanisms to prevent this undesirable influence which may bias the study. A cautious engagement with the more influential stakeholders has been suggested as an important strategy starting from protocol development stage. In addition, early development, (at or before the project inception phase) of clear standard operating procedures (SOPs) for having an effective transparent decision-making process that includes listening to all stakeholders is recommended. The research team should show how the SOPs will be widely distributed and made easily accessible to all stakeholders to prevent biases arising from lack of transparency, and conflict of interest. All research team members should receive on the job training on responsible conduct of research oriented to the needs of the antibacterial trial.

### The trial design affects how RECs evaluate ethical challenges in MDA

The main-stay study designs in antibiotic MDA trials are pragmatic randomised clinical or community trials, although there is a move to alternative trial designs: conventional cluster randomised trials (CRTs) and increasingly step-wedge and adaptive CRTs. The latter offer a number of potential advantages, including being more efficient in combining intervention delivery with implementation science and making the trial process more socially acceptable. All study designs have particular ethical considerations.

The CIOMS guidelines [26] and the Ottawa statement [28] on the ethical design and conduct of CRTs provide guidance on the use of conventional CRTs and alternative trial designs. Recent literature has highlighted the gaps in the Ottawa statement and called for its revision to cater for the unique needs of LMICs [28–31] which are relevant for REC reviews.

The RECs should be alert to the challenging design-related ethical issues (Table 1). They should provide appropriate initial review of the protocol and during study progress to ensure the trial’s scientific validity or integrity [4, 69–71]. The key concerns are the ethical issues associated with conventional CRTs as identified in the CIOMS guidelines [26] and in the Ottawa Statement [43]: justification of the specific design; identification of research participants; obtaining informed consent; roles and authority of gatekeepers; assessing benefits and harms; and protecting vulnerable participants. There is increasing recognition of the ethical issues associated with conventional CRTs [43, 53, 72–74] and other alternative trial designs, including step-wedge and adaptive trials [2, 3, 6, 70, 71, 75–81]. The latter assess the accumulating data and based on interim analysis results make modifications to the trial’s structure or parameters. From an ethics perspective, these designs complicate applying equipoise [3], are more prone to bias, expose more people to harm than conventional RCTs, increase the difficulty of risk–benefit evaluations, and complicate discussions of risks with participants. RECs must be aware of these issues.

The units of randomization, intervention, and outcome assessment may also differ—sometimes, in the same trial. There is need for close attention to CRTs with a fixed number of clusters that have large sample size per cluster. A CRT that has large clusters with the numbers of participants per cluster exceeding those required to address the main study aim(s) is ethically problematic. The excess participants do not add social value despite their exposure to risks and burden of participation [73]. However, a reasonable justification is the prior identification of large cluster sizes and prespecifying fully powered secondary analyses [73].

Evidence is accumulating about the unique ethical challenges experienced in the design and conduct of CRTs [31, 73, 82–87] and other alternative trials. However, there are relatively few articles addressing specific ethical issues arising in LMIC settings [29, 88]. These ethical challenges may be magnified by contextual factors peculiar to LMICs [29–31, 72]. For example, the REC members need to have a good understanding of moral status or hierarchies within social groups especially for purposes of obtaining community and individual consent.

The CRT design often includes pragmatism [74, 89], and pragmatic trials have an added layer of ethical challenges [90, 91]. Pragmatic trials are primarily used to determine the effects of an intervention under the usual conditions it will be applied [92]. The key value add of pragmatic trials is to inform policy decisions by providing evidence for adoption of the interventions into public health practice [92]. Thus, MDA trials utilise a public health mode of intervention delivery.

As a result of their typical design, RECs should review these MDA studies as public health (population) intervention trials and not as clinical trials. Only by doing so can RECs fully appreciate the ethics challenges of MDA research. By using an intervention already accepted (antibiotics), it can be challenging for the REC to determine whether the control (comparator) arm should receive usual locally available or augmented care—a challenge well recognized in AIDS research in LMICs in the 1990s [88, 93, 94]. Determining what constitutes usual care may be extremely challenging, but the REC has the obligation to provide guidance and justification based on the local context for what intervention should be provided in the control arm. The decision has important ethical implications including: (a) a need to assure maintenance of scientific equipoise, i.e., genuine uncertainty or sufficient disagreement within the expert community of professionals about the relative merits of the interventions across the different study arms of a controlled trial [69], (b) what evidence to use in defining a trial intervention or its comparator as minimal risk, (c) what is sufficient justification for alterations and waivers of informed consent from participants, and (d) which risks, burdens and benefits need to be disclosed to potential participants in the consent process in the context of stated standard, or usual care [3]. The easy access to antibiotics by the general public in the absence of a prescription in many LMICs may create an added ethical problem of contamination and minimising the intervention effectiveness.

These unique design considerations mean RECs should be clear on who is a research participant in this trial [28]. Their correct identification is essential for conducting a proper analysis of benefits and harms and for informed consent. The study design should include obtaining community consent from recognised legitimate and trusted authority. Clarity is essential for determining who is a research participant, and how or when should informed consent be obtained from them. RECs also need to explore risks and burdens beyond the obvious for example, to determine whether the personnel who deliver the medication may be exposed to certain risks like carriage of resistant bacteria (if AMR is a problem) and thus deserving of protection. Processes should be developed in the communities for monitoring access, transparency, accountability, and protecting the rights of the disadvantaged and vulnerable individuals (to the interventional drug(s).) [95–97].

### Improving REC review of MDA studies

Antibacterial MDA trials raise challenging ethical issues [22]. Existing evidence suggests REC members’ inadequacy in evaluating some research protocols especially those employing newer study designs and methods [4, 8, 98]. This leads us to suggest a continuing need to enhance REC capacity to become fit-for purpose as summarised in Table 2. We suggest two key strategies.

**Table 2:** Collaboration between local and foreign RECs/IRBs

Domain	Areas of collaboration	Mechanisms of collaboration	Implementation of the collaboration
Collaboration between RECs	Development of plans for the local and foreign RECs to collaborate on MDA studies	Improved bilateral communication about MDA review findings between the LMIC and HIC RECs/IRBs Joint REC meetings (e.g., virtually) Sharing REC capacity building resources	Guiding documents for collaboration are in place To be agreed upon by LMIC, HIC REC/IRBs Appropriate resources for LMIC REC to be identified by both RECs
	Resolution of conflicting decisions	Development of joint resources, such as SOPs to manage any conflicting reviews of the MDA study	Existence of SOPs to manage conflicting reviews
	Plans for evaluation of the success of a collaboration	Joint evaluation of the MDA review process by both HIC and LMIC RECs/IRBs	Sharing ongoing evaluation successes, challenges, and lessons learned

*HIC* High-income country, *IRB* Institutional Review LMIC, *LMIC* Low- and middle-income countries, *MDA* Mass drug administration, *REC* Research Ethics Committee, *SOPs* Standard operating procedures

The first is for RECs to engage external experts with expertise for addressing the ethical concerns of antibacterial MDA trials and are familiar with social-cultural, political and economic context of the study site(s). This is especially critical at the initial ethics review, which provides an ex-ante assessment to ensure the ethical validity of a trial design and the planned processes, protocol adequacy and other documents prior to approval and to meet applicable regulatory requirements. Meaningful reviews give researchers opportunities to receive useful feedback from the RECs. This may improve the research quality and increase public trust in the research [99]. RECs should consider training their members regarding the research ethics related to antibacterial MDA research, and pragmatic cluster randomized trials including the newer alternative designs.

Second, since these trials are usually sponsored by high-income countries (HICs) the research proposals should be reviewed as a collaborative partnership by both HICs and LMICs RECs or IRBs. This will contribute to bridging the knowledge and skills gap (between HIC and LMIC RECs) through bidirectional learning and the enhanced collaboration. The separate reviews may provide differing recommendations and outcomes. This divergeance will provide opportunity for increased dialogue. All too often, these RECs from different contexts operate in isolation, with some institutions requiring LMIC review first (or vice versa). Now more than ever, online and face-to-face collaboration between LMIC and HIC RECs are possible to augment and complement each other through joint reviews [100], sharing minutes of ethical review meetings, having joint technical resources and capacity building activities. Still, the local REC takes priority, and can make significant contribution to ethical review using their familiarity of the social-cultural, political and economic context of the study host country.

In the absence of appropriate local research guidelines, the LMIC REC reliance on the international ones for which they may not have expertise to interpret and appropriately apply may be a challenge. However, the HIC REC can fill the gap by sharing their ethics expertise, and available web-based educational ethics materials with LMIC. The former would provide widely accessible resources, and its members would commit time to support the review processes and even consult their wide network of colleagues, on the ethics and science of research.

A possible limitation is that because we only searched the English literature, we may have missed some articles that addressed our topic of interest. Research involving mass antibiotic administration intervention is relatively recent resulting from the potential use of azithromycin in children mainly to reduce mortality and respiratory infections. There is currently insufficient literature specific to the REC review of antibiotic MDA studies to allow for a systematic review.

## Conclusions

We have identified four domains of ethical issues (social value, assessment of risks and burdens, stakeholder engagement and ethical issues associated with study designs) that require increased attention by RECs during initial and continuing review of MDA research. Our review calls upon RECs to broaden their assessment of MDA research along each of these four domains by systematically assessing social value in comparison to other possible interventions; to assess risks that are both short- and long-term, within and beyond the research; to engage all stakeholders, not just communities; and to consider those unique issues associated with typical MDA study design. Empirical research is needed to address the gaps outlined in this paper to produce evidence-based guidance frameworks to facilitate ethical reviews that will arrive at ethically and methodologically sound decisions. This work is the first step in the development of an evidence-based guidance framework to strengthen RECs regarding review of antibiotic MDA trials. Finally, we encourage the emergence of strong collaborations between RECs in LMICs and those in HICs for mutual capacity building.

## Data Availability

The literature used and referenced in this study is all available in peer reviewed journals and is publicly accessible.

## Abbreviations

ADR: Adverse drug reactions
AMR: Antimicrobial resistance
AVENIR: Azithromycin Pour la Vie Des Enfants au Niger—Implémentation et Recherche
CIOMS: Council for International Organizations of Medical Sciences
CRT: Cluster randomised trial
HIC: High income country
IRB: Institutional Review Board
LAKANA: Large-scale Assessment of the Key health-promoting Activities of two New mass drug administration regimens with Azithromycin
LMICs: Low- and middle-income countries
MDA: Mass drug administration
MORDOR: Macrolides Oraux pour Réduire les Décès avec un-Oeil sur la Résistance
REC: Research Ethics Committee
SAFE: Surgery antibiotics face cleanliness and environmental improvement
SANTE: Sauver Avec l'Azithromycine en Traitant Les Femmes Enceintes et Les Enfant
SMC: Seasonal malaria chemoprevention
SOP: Standard operating procedures
TV: Television
WHO: World Health Organisation
